# Jejunal Gossypiboma Mimicking a Gastrointestinal Stromal Tumor: A Case of a Rare Iatrogenic Complication and Review of the Literature

**DOI:** 10.7759/cureus.75651

**Published:** 2024-12-13

**Authors:** Michail Angelos Papaoikonomou, Europi Michailidou, Christos Pogiatzis, Maria Eleni Michailidi, Gregorios Panselinas

**Affiliations:** 1 Department of General Surgery, General Hospital of Thessaloniki Agios Pavlos, Thessaloniki, GRC; 2 Department of Pathology, General Hospital of Thessaloniki Agios Pavlos, Thessaloniki, GRC

**Keywords:** cesarean section, exploratory laparotomy, gastrointestinal stromal tumor (gist), gossypiboma, surgical oncology

## Abstract

The term "gossypiboma" or "textiloma" is used to describe a mass of retained cotton matrix and the surrounding inflammatory response inside a body cavity following surgical intervention. The precise incidence of this phenomenon is uncertain, as cases are frequently underreported due to concerns related to medical malpractice. This rare complication poses a diagnostic dilemma due to the non-specific clinical and radiological features, which can result in significant morbidity and mortality. While diagnosis of this condition has been reported through various imaging modalities such as abdominal ultrasound and computed tomography, there are instances where cases do not present with typical findings, which can make it tough to make a preoperative diagnosis. The differential diagnosis from submucosal tumors of the gastrointestinal tract becomes challenging, particularly when the tumors are in contact with the gastrointestinal tract. It is possible that patients may be incorrectly informed that masses may be malignant, which could result in unnecessary extirpative surgery. In some cases, the diagnosis is discovered postoperatively. This case report presents a case of a 42-year-old woman who was initially suspected of having a gastrointestinal stromal tumor based on radiological findings. However, the patient was ultimately diagnosed with a gossypiboma following surgical intervention. A gross examination of the surgical specimen revealed that a gauze left over from a cesarean section performed nearly 20 years ago was the source of the gossypiboma. It is therefore evident that meticulous counting of surgical instruments and materials is crucial for patient safety and optimal outcomes.

## Introduction

Gossypiboma derives from the Latin word "gossypium" (cotton) and the Swahili word "boma" (place of concealment). Originating from the Greek term "oma," (illness, tumor, or swelling) and the Latin word "textilis," (weave in) comes the term "textiloma" [1]. According to the current literature, 0.02% of procedures result in the uncommon complication of retained surgical materials and 70% of these are surgical sponges [2]. It is estimated that one in 100 to 3000 surgical procedures will result in the inadvertent retention of a surgical swab, with associated morbidity of 11-35%, correlating with the size and duration of the retained foreign body [1,3,4]. At one in 1000 to 1500 cases, the prevalence is high for intra-abdominal procedures [1,3]. A comprehensive history of the patient, a physical examination, and laboratory and radiological investigations can all contribute to reaching an accurate diagnosis. The recommended course of treatment is surgical exploration and resection of the mass. Herein, we present a case of a retained gauze following a cesarean section performed nearly 20 years ago that was misdiagnosed as a gastrointestinal stromal tumor (GIST) and a brief review of the existing literature.

## Case presentation

**Figure 1:**
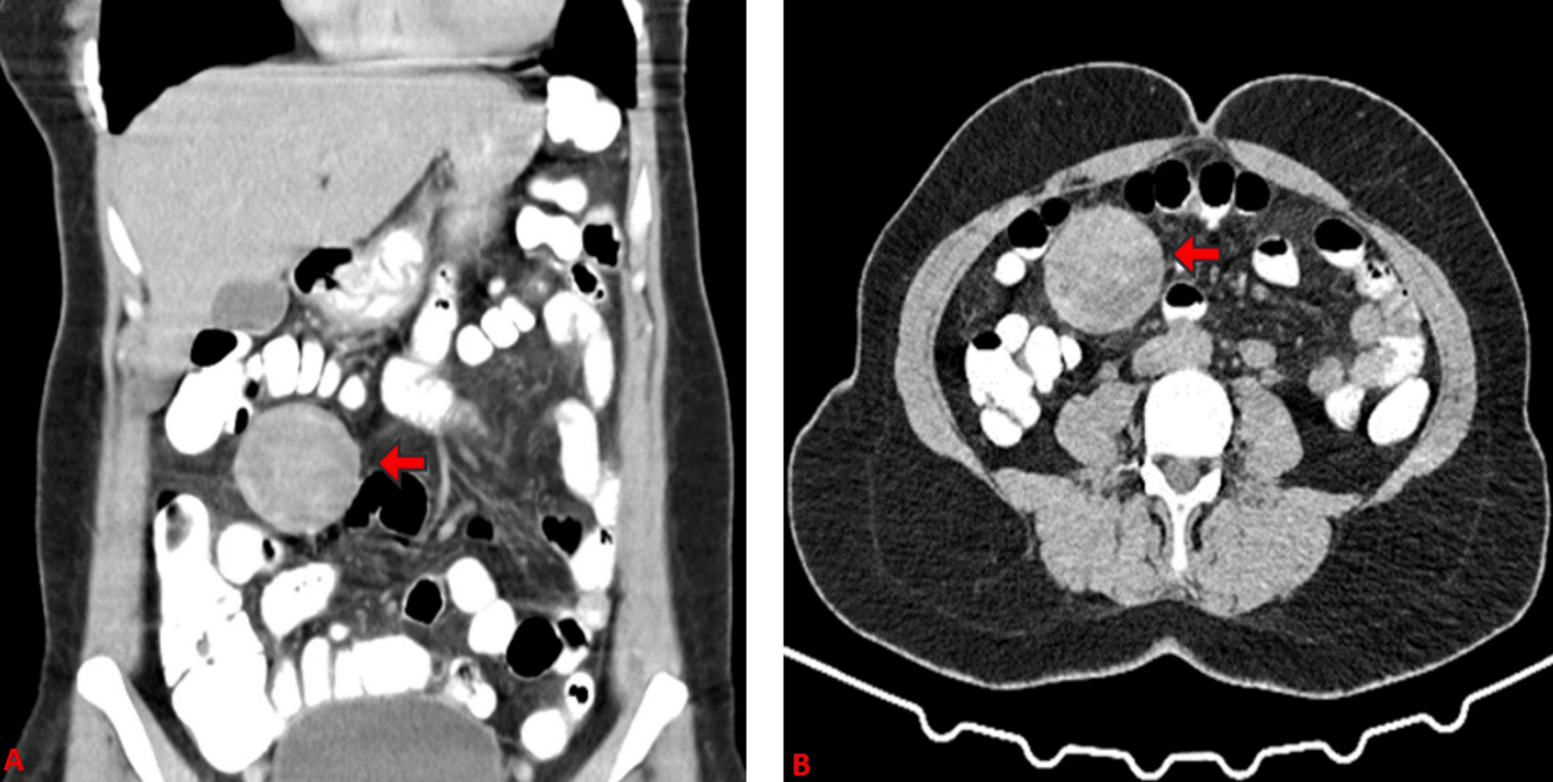
CT of the abdomen coronal view (A) and axial view (B) indicating a spherical mass (5.4 x 5 cm) with a thick enhanced wall after intravenous contrast (red arrows). The mass is located in the larger omentum in close relation with the jejunum with no sign of obstruction or intestinal ischemia.

A 42-year-old woman presented to the emergency department with a gradual onset of abdominal distension, mild abdominal pain, nausea, and frequent episodes of abdominal discomfort for nearly two years, which was exacerbated in the last few hours. No fever or sudden weight loss was noted and the patient was stable on examination (heart rate was 90 beats per minute, blood pressure was 120/77mmHg, and oxygen saturation was 98%). The abdomen was distended with mild generalized tenderness with bowel sounds present, but no guarding or rigidity was observed. Superficial and deep palpation revealed no abnormalities. She had previously undergone two cesarean sections (20 and 24 years ago). A complete blood count showed white blood cells at 13.5 x103/μL, neutrophils at 81%, hemoglobin at 11.6 g/dL, and C-reactive protein at 4.4 mg/dL. An upright abdominal X-ray was normal without significant air-fluid levels. Urgent CT with contrast was ordered in cooperation with the internal medicine department to determine the exact origin of the patient's symptoms, decide the proper management of this patient, and exclude any acute abdominal pathology. CT scan showed a round, well-defined mass with a dense central part and a thick enhancing wall in proximity to the jejunum resembling a GIST (Figure 1).

**Figure 2:**
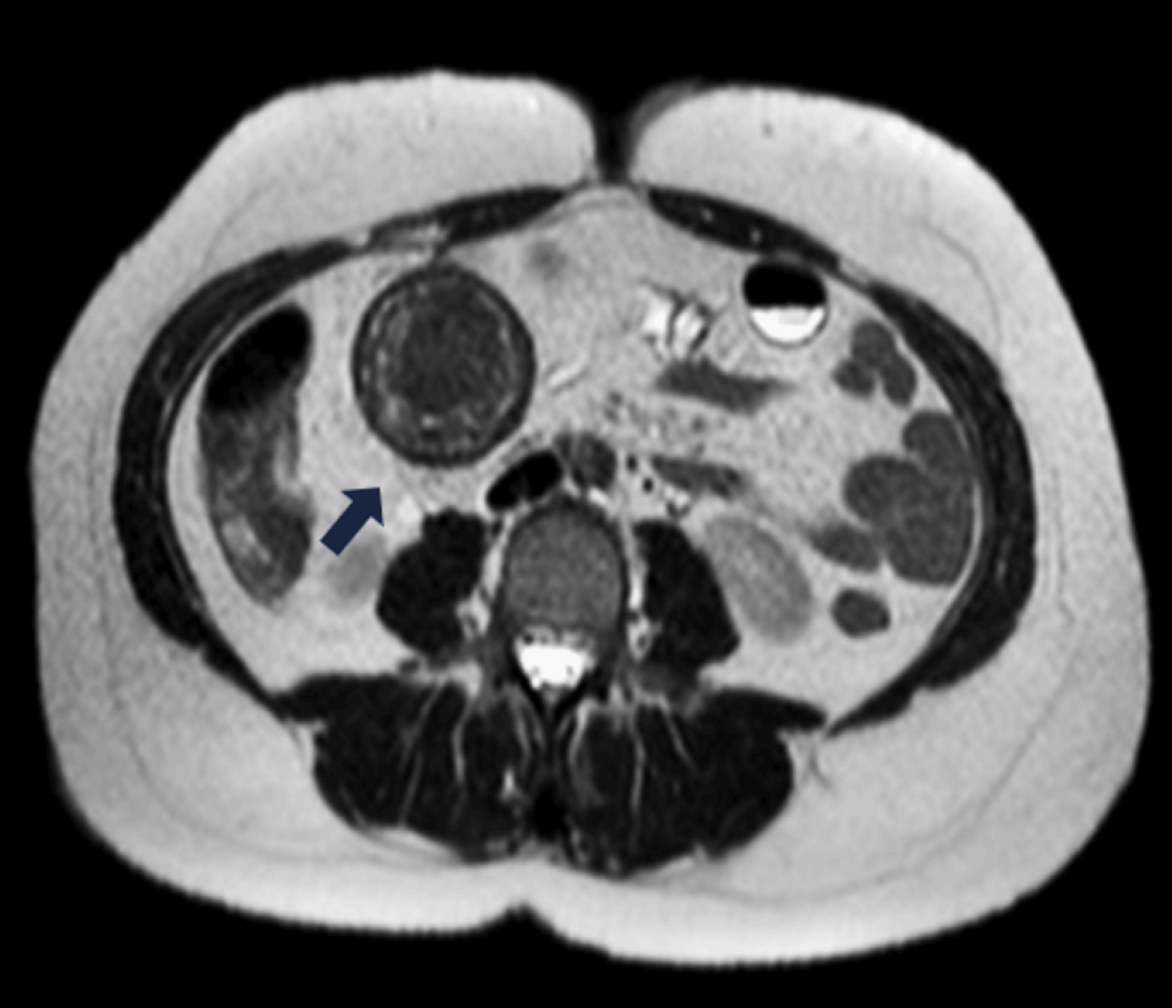
T2-weighted MRI of the abdomen revealed a distinct heterogeneous mass (black arrow) with peripheral enhancement and low signal following IV contrast administration.

The patient was initially managed with intravenous fluids and empirical treatment with broad-spectrum antibiotics was initiated due to elevated inflammatory markers (cefuroxime 750 mg, 1 x 3). To gain further insight into the mass and its origin, an MRI scan was conducted. An MRI scan revealed the presence of a prominent, spherical subhepatic mass in direct relation to the small bowel loops. In addition, the mass exhibited heterogeneity and low signal on both T1 and T2 sequences with peripheral enhancement, following the administration of intravenous contrast. According to these findings, the radiologist concluded that this mass can be attributed initially to either a GIST or a desmoid tumor (Figure 2).

**Figure 3:**
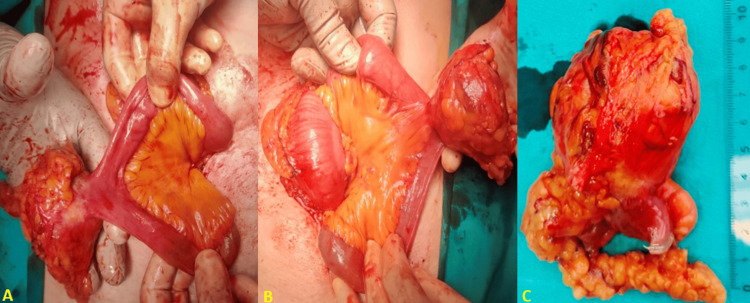
A & B: Intraoperative photographs after adhesiolysis showing the encapsulated mass adherent to the jejunal serosa. C: The surgical specimen demonstrated a 5.4 x 5 cm spherical fibrinous mass adherent to the jejunal wall.

Due to the location of the mass and its proximity to the small intestine, ultrasound-guided biopsy or molecular testing (e.g., KIT and PDGFRA) was not an option for the histopathological confirmation of the original diagnosis and was not considered prior to surgical intervention in this hemodynamically stable patient for safety reasons. On the second day of admission, the patient was subsequently transferred to the operating room for an exploratory laparotomy. The patient was anesthetized with general anesthesia and intubated, and a midline incision was made. Intraoperatively, a white-grey mass with a firm consistency, measuring 5.4 × 5 cm, was found to be attached to the jejunum with serosal involvement (Figure 3).

**Figure 4:**
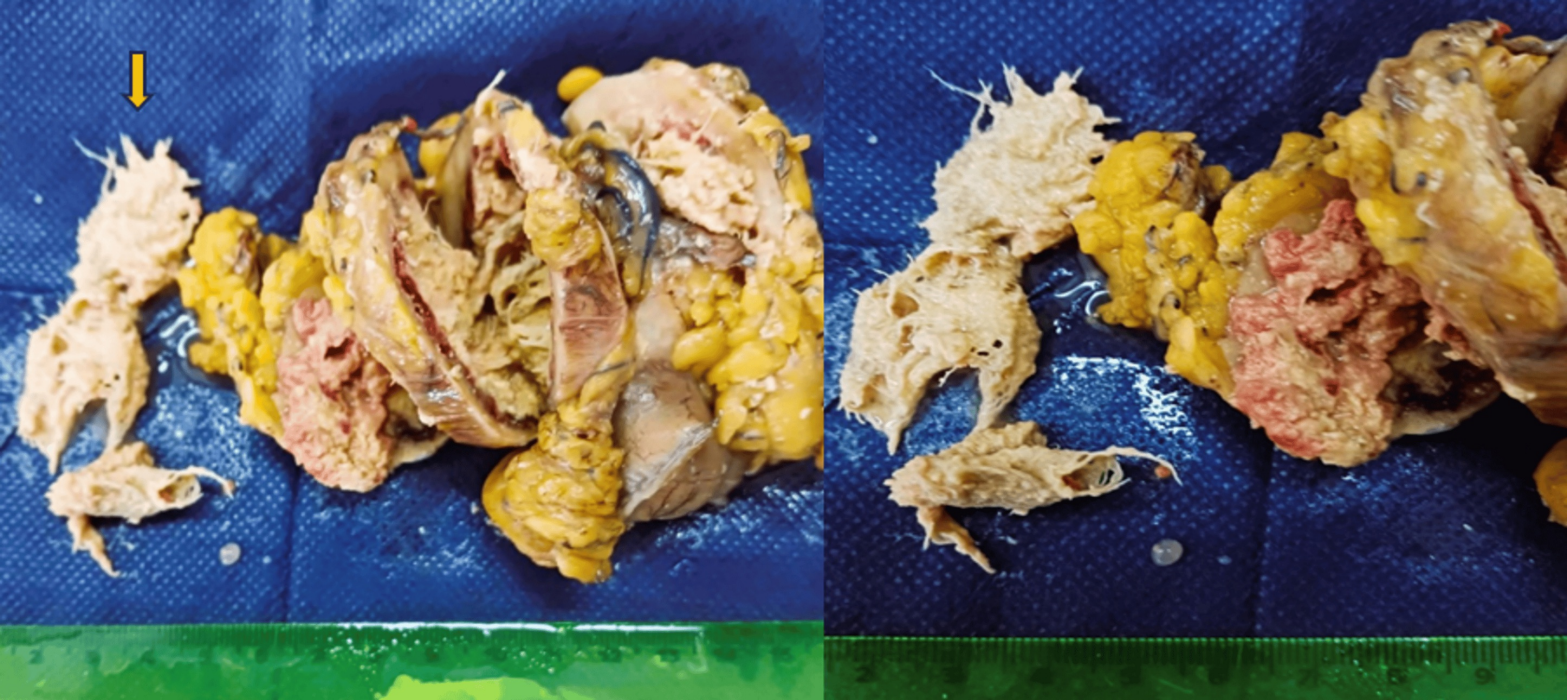
A gross examination of the surgical specimen showed a 3 x 5.4 x 5 cm spherical fibrinous mass adherent to the jejunal wall, characterized as a gossypiboma containing a retained gauze (yellow arrow).

Adhesions were released, complete mobilization of the mass was achieved, the affected part of the jejunum was removed with a wide surgical margin and primary side-to-side anastomosis was performed. Drains were placed near the anastomosis and at the pelvic cavity and the wound was closed in layers to acquire better safety and effective monitoring for a possible anastomotic leak. Upon gross examination, the specimen was sliced into fragments and a surgical gauze was visible (Figure 4).

**Figure 5:**
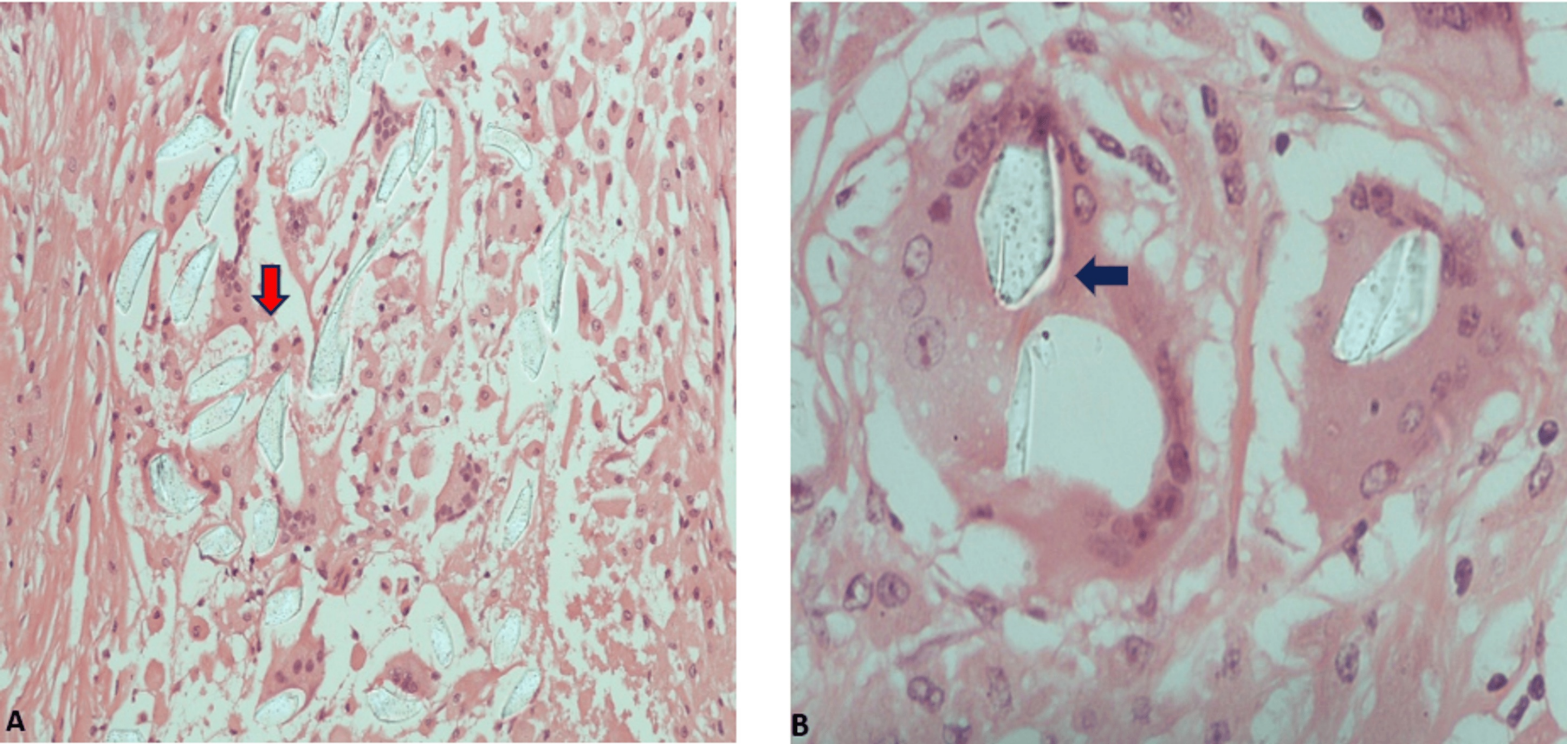
Biopsy specimen. (A) Low power magnification (x10) and (B) high power magnification (x40) showing foamy multinucleated histiocytes (blue arrow) surrounding the birefringent material (red arrow) (hematoxylin and eosin stain).

Histopathology of gossypiboma’s fibrinous capsule showcased abundant foamy histiocytes, melanin-gray pigment, and segments of birefringent material (foreign body) with focal inflammatory reaction by giant cells. Multinucleated giant cells (MGCs), which were polykaryons of monocytic origin, were in close proximity to foreign bodies and the immune system's response to them. Inside the capsule, there was plenty of necrobiotic material surrounding segments of the foreign body. Immunohistochemistry exhibited CD68+ foamy histiocytes on the capsule of the mass. No signs of malignancy were observed during gross examination (Figure 5).

In the postoperative course, the patient was kept nothing by mouth (NPO) for four days and received intravenous fluids for four days and antibiotics for six days according to the instructions of the attending doctor. The patient was stable on follow-up and the drains were removed on the fifth day. The patient was discharged on the seventh day and remained stable during follow-up.

## Discussion

Gossypibomas, initially diagnosed as intra-abdominal tumors, can be a complication from any surgery, with a higher incidence in previous obstetric and gynecological procedures, mostly cesarean sections, open cholecystectomy, gastrectomy, and urinary operations [5]. It affects women more frequently than men and is higher in developing nations where emergency surgeries during the night are supported by an epileptic power supply [6]. Risk factors for surgical materials’ retention include emergency surgery, high body mass index, prolonged procedures, inexperienced medical staff, disorganization/miscommunication, and unanticipated changes to the surgical technique or team [7]. Following surgery, two biological responses can occur: an inflammatory response resulting in an abscess (exudative gossypiboma) or a non-infectious response in which the foreign body is encased in fibrous tissue (fibrinous gossypiboma). The first reaction is septic and usually symptomatic, requiring urgent surgical intervention. The second type is an aseptic reaction, which can remain silent throughout the years, leading to the formation of a capsule that serves as a shield between the foreign material and the tissues around it and adhesions [8]. A surgical swab that is still in place can eventually shatter or fragment, releasing debris and escalating inflammation [9]. In our case, the second type of reaction occurred. The most commonly affected sites are the abdomen (56%), the pelvis (18%), and the thorax (11%), but gossypibomas have also been reported in the extremities, central nervous system, breast, head, and neck region [1].

Clinical manifestations depend on the location and the level of bacterial contamination of the retained item, with approximately one-third of patients exhibiting no symptoms and discovering the condition by chance [4]. Symptoms range from moderate abdominal pain, abdominal distention, constipation, nausea, vomiting, and chronic pain from adhesions to serious complications [10]. Early complications include infection, hemorrhage, dehiscence, and delayed healing of the wound [4,8]. Our case was presented with an exacerbation of chronic abdominal pain and distress for which she sought medical advice for the first time. Transmural migration of an intra-abdominal gossypiboma has been reported, resulting in the expulsion of the sponge through the rectum and mechanical intestinal obstruction [9]. Other late complications include adhesions, abscesses, fistulas, malabsorption syndrome, peritonitis, and sepsis [4]. The duration between the causative operation and presentation with retained surgical material ranges from a few days to 40 years, with the peak occurring within the first two months post surgery [11]. After a cesarean section, the retained material usually moves to the rectum and the sigmoid colon, based on the anatomic location of each procedure [12]. The diagnosis of our case was established after over 20 years from the primary operation without knowing which cesarean section accounts for it.

Diagnostic imaging studies can confirm the diagnosis of this challenging entity, with only one-third of cases having the correct preoperative diagnosis. Plain radiography is most frequently used to detect gossypibomas whereas CT scan seems to be more reliable [1]. The presence of fine linear radio-opacity of textile material is usually an indicator of retained surgical gauze. The absence of the radiopaque marker thread on plain radiography cannot completely rule out gossypiboma because it can come off, get distorted, or disintegrate over time [13]. According to the existing bibliography, calcifications formed over time can be depicted as a “calcified reticulate rind sign” mostly on CT images, which could be a helpful feature in identifying this pathology [14]. In our case, the X-ray was normal, and the foreign body was missed. A cystic structure with intense posterior shadowing, a hypoechoic ring, a hyperechoic wavy striped structure, and complex mass are characteristic patterns seen in ultrasonography [4]. On CT scans, gossypiboma can mimic soft tissue tumors, hematomas, abscesses, or even cystic lesions [15]. Particularly in long-standing cases, it is difficult to distinguish gossypiboma from an intra-abdominal abscess or tumor on CT, as air bubbles, calcification of the cavity, and contrast enhancement of the capsule may be seen in all these conditions [4]. In addition, the presence of a distinct feeder artery has been referred to as a characteristic that could lead to the diagnosis of a malignant tumor on CT images [16]. Our case is different since the mass mimicked a GIST in the CT scan and air bubbles or calcification were absent. On MRI, gossypiboma is characterized as a well-defined, capsulated soft tissue mass with a low signal on T1 and a high signal on T2 images with a whorled or spongiform pattern and peripheral enhancement of its wall after intravenous contrast administration [17]. In our case, MRI revealed a well-circumscribed mass with enhancement of its wall and low signal on both T1 and T2 sequences. Based on these findings, the differential diagnosis included a jejunal GIST or a desmoid tumor of omentum. GIST is the most frequent misdiagnosis among gossypibomas that mimic tumors [5]. It is important to suspect gossypiboma if a patient has had prior surgery when CT or MRI results show signs of GIST [18]. In the case of an intra-abdominal mass, the use of percutaneous biopsy guided by ultrasound or CT and histopathologic examination to rule out a malignant tumor should also be highlighted [19]. Lastly, endoscopic ultrasound fine-needle aspiration in the preoperative diagnosis of intra-abdominal gossypiboma has also been reported once with unknown safety [20].

Surgical excision is typically performed through explorative laparotomy, with endoscopic or laparoscopic methods being an option, if the retained material is detected early. If the object has been retained for years, there is a great chance of erosion or fistula formation, which is the main reason that an open approach is preferred in these chronic cases [1]. Dense adhesion typically forms around the gossypiboma as a result of the disease's chronicity and severe foreign body reaction. Severe intestinal damage was reported in about 30% of cases, necessitating part colectomy [9]. In these situations, the tumor cannot be removed without also harming the intestine due to the extremely thick adhesions, dictating the removal of a portion of the intestine as well [9]. In this patient, exploratory laparotomy with resection and anastomosis of the jejunum was decided due to the primary suspicion of a GIST based on imaging findings. En block dissection of the involved portion of the jejunum was executed due to adhesion formation and partial penetration of the mass into the intestinal wall.

Prevention of this iatrogenic complication is very important. Legal claims for gossypiboma are likely to be successful, seriously harming the reputation of the doctor, the medical profession, as well as the medical facility where the initial surgery was carried out. To prevent this, surgical teams should count sponges and instruments at various stages of the procedure (prior to the procedure beginning, when a new item is added, when a cavity is closed, during wound closure, and during skin closure), and search for missing items if discrepancies are found [9]. Simple precautions like maintaining a complete pack count, marking packs with markers, and taking postoperative abdominal radiographs can reduce the gossypiboma’s incidence. Newer technology like computer-aided diagnosis software and radiofrequency identification could also reduce foreign body retention, but more studies are needed to evaluate their effectiveness [2,8]. In Table 1, we present a synopsis of gossypibomas masquerading tumor cases mentioned in the article.

**Table 1:** Clinical information of the included case reports of intra-abdominal gossypibomas that mimicked tumors. US: ultrasound; CT: computed tomography; MRI: magnetic resonance imaging; GIST: gastrointestinal stromal tumor.

Author	Year & country	Age	Causative operation	Interval	Symptoms	Imaging	Initial diagnosis	Treatment
Yamamura et al. [16]	2008, Japan	78	Partial gastrectomy, cholecystectomy	15 or 40 years	Asymptomatic	US, CT, and MRI	Gastric GIST	Surgical resection
Cheon et al. [14]	2011, Korea	78	Partial gastrectomy	30 years	Acute epigastric pain	US, CT and esophagogastroduodenoscopy	Gastric GIST	Resection of the gastric fundus
Kawamura et al. [18]	2012, Japan	41	Cesarean section	2 years	Recurrent abdominal pain	CT and MRI	GIST	Laparotomy, enterectomy, and anastomosis
Celik et al. [4]	2021, Turkey	36	Ectopic pregnancy	7 years	Severe abdominal pain and distention	X-ray, US, and CT	Soft tissue mass	Laparotomy, enterectomy, and anastomosis
Menkiti et al. [6]	2021, Nigeria	31	Cesarean section	1 year	Growing left lumbar lump with intermittent low-grade fever	US	Tumor of the transverse colon	Laparotomy, enterectomy, and anastomosis
Han et al. [5]	2023, China	33	Cesarean section	7 years	Pain, distention, nausea, vomiting	CT	Jejunal tumor	Laparoscopic resection and jejunojejunostomy
Rhoul et al. [17]	2024, Morocco	48	Open cholecystectomy	12 years	Abdominal pain	CT	Mesenteric tumor	Laparotomy, partial removal of the jejunal wall, and suture of the jejunum
Dainaka et al. [20]	2024, Japan	63	Distal gastrectomy	44 years	Asymptomatic	US, CT, and ultrasound endoscopy	GIST	Tumor resection and splenectomy

## Conclusions

In conclusion, although gossypiboma is a rare and challenging condition to diagnose, it is preventable. Gossypiboma should be included in the differential diagnosis of any patient with a history of prior surgery who presents with non-specific complaints, abdominal pain, wound breakdown, or a soft tissue mass. It is imperative that all possible measures be taken to prevent this condition, as it can result in significant complications, additional costs, and serious medico-legal implications. Adherence to established safety protocols and effective communication among the surgical team can help reduce the incidence of this pathology. Further research on optimal surgical environments and communication strategies can contribute to the minimization of this devastating complication.
